# From virtual pregnancy to digital twin obstetrics: multimodal data integration for personalized prediction of pregnancy complications

**DOI:** 10.3389/fmed.2026.1867368

**Published:** 2026-06-18

**Authors:** Mina Xu, Lijun Ruan, Xin Xu, Haiou Qi

**Affiliations:** 1 Nursing Department, Sir Run Run Shaw Hospital, Zhejiang University, School of Medicine, Hangzhou, Zhejiang, China; 2 Department of Traditional Chinese Medicine, The Fifth Affiliated Hospital of Southern Medical University, Guangzhou, Guangdong, China

**Keywords:** digital twin, machine learning, multimodal data integration, obstetrics, precision medicine, pregnancy complications, virtual pregnancy

## Abstract

Pregnancy complications, including preeclampsia, gestational diabetes mellitus, preterm birth, fetal growth restriction, and placental insufficiency, remain major contributors to maternal and neonatal morbidity worldwide. Conventional prediction models in obstetrics have traditionally relied on static clinical variables collected at discrete gestational time points. Although these approaches are clinically convenient, they are insufficient to capture the dynamic, heterogeneous, and nonlinear nature of pregnancy physiology. Recent advances in computational modeling, artificial intelligence, biomedical data acquisition, and continuous physiological monitoring have introduced the concepts of virtual pregnancy and digital twin obstetrics. Virtual pregnancy modelsaim to simulate maternal–placental–fetal interactions through mechanistic or computational frameworks, whereas digital twins aim to extend this concept by integrating real-time multimodal data streams to construct continuously updated, patient-specific models. This review discusses the conceptual transition from virtual pregnancy to digital twin obstetrics and examines how multimodal data integration can support personalized prediction of pregnancy complications. Particular attention is given to clinical phenotyping, obstetric imaging, radiomics, multi-omics data, wearable-derived physiological monitoring, and machine learning–based modeling strategies. The review further evaluates potential applications in preeclampsia, gestational diabetes mellitus, preterm birth, fetal growth restriction, and placental dysfunction. Although digital twin obstetrics represents a promising but still largely conceptual framework for precision maternal–fetal medicine, major challenges remain, including data heterogeneity, limited external validation, model interpretability, ethical governance, privacy protection, and clinical implementation. Future work should prioritize standardized data infrastructures, prospective multicenter validation, explainable artificial intelligence, and clinically interpretable decision-support systems.

## 1 Introduction

Pregnancy is not a static biological condition but a temporally evolving physiological process characterized by coordinated adaptations across cardiovascular, endocrine, metabolic, immune, and placental systems. These adaptations are necessary to support fetal growth and maintain maternal homeostasis. However, when regulatory mechanisms fail or maladaptive responses emerge, pregnancy may progress toward pathological states, including preeclampsia, gestational diabetes mellitus, preterm birth, fetal growth restriction, and other placenta-mediated complications. These disorders are not isolated clinical events; rather, they often reflect gradual disturbances in maternal–placental–fetal communication that begin before overt clinical symptoms become apparent (1–3).

A central difficulty in obstetric care lies in the early identification of individuals who are likely to develop complications. Traditional risk assessment models are usually based on demographic characteristics, obstetric history, routine laboratory markers, blood pressure, body mass index, and ultrasound measurements. These variables are useful and remain indispensable in clinical practice, but they often provide only a partial representation of pregnancy biology. Many conventional models are cross-sectional, population-derived, and limited to single time-point prediction. Consequently, they may perform inadequately in heterogeneous populations and may fail to reflect the dynamic progression of disease (4, 5).

The limitations of static prediction are especially evident in disorders such as preeclampsia and preterm birth. Preeclampsia may involve abnormal placentation, angiogenic imbalance, endothelial dysfunction, inflammatory activation, and maternal cardiovascular maladaptation. Preterm birth may arise from infection, inflammation, cervical insufficiency, uterine overdistension, vascular pathology, or stress-related neuroendocrine mechanisms. Gestational diabetes mellitus is similarly heterogeneous, involving maternal insulin resistance, pancreatic *β*-cell compensation, adipose inflammation, and metabolic reprogramming. Because these disorders arise from interacting biological pathways, prediction based on a small number of conventional clinical variables is unlikely to be sufficient. Evidence from Mendelian randomization linking homocysteine with polycystic ovary syndrome further illustrates how endocrine-metabolic reproductive disorders can be examined through causal frameworks (6).

From a life-course perspective, causal analyses of pregnancy and menstrual history in relation to endometrial cancer also suggest that reproductive events may leave measurable metabolic and disease-risk signatures beyond the index pregnancy (7).

In recent years, the rapid development of artificial intelligence, biomedical informatics, high-throughput molecular profiling, advanced imaging, and wearable devices has provided new opportunities for obstetric prediction. The concepts of virtual pregnancy and digital twin obstetrics have emerged from this technological context. Virtual pregnancy refers broadly to computational representations of pregnancy-related biological processes, including maternal physiology, placental function, fetal growth, and disease trajectories. Digital twin obstetricsaims to go further by constructing a dynamic digital representation of an individual pregnancy that could be updated using real-time or longitudinal data (8–10).

This transition marks an important shift in obstetric thinking. Rather than asking whether a pregnant individual belongs to a high-risk group based on population averages, digital twin obstetrics seeks to estimate how that individual’s risk profile evolves over time. In principle, such a system could integrate electronic health records, ultrasound images, Doppler parameters, laboratory biomarkers, omics data, continuous glucose monitoring, heart rate variability, activity patterns, and environmental exposures into a continuously updated model. This model could then support early warning, risk stratification, intervention simulation, and individualized management.

However, enthusiasm for digital twin obstetrics must be balanced with methodological caution. Many existing artificial intelligence models in obstetrics remain retrospective, single-center, insufficiently validated, and difficult to interpret. Digital twin systems require not only advanced algorithms but also high-quality longitudinal data, standardized definitions, transparent reporting, clinical interpretability, and ethical governance. Therefore, the goal of this review is to present a structured overview of the field, focusing on the conceptual development from virtual pregnancy to digital twins, the role of multimodal data integration, modeling strategies, clinical applications, current limitations, and future directions (11–13).

## 2 Conceptual evolution from virtual pregnancy to digital twin obstetrics

Virtual pregnancy models were initially developed within the broader framework of systems biology and computational physiology. Their purpose was to represent specific aspects of pregnancy through mathematical, mechanistic, or simulation-based models. Early approaches often focused on uteroplacental circulation, fetal growth, oxygen transport, hormonal regulation, or maternal cardiovascular adaptation. These models were valuable because they allowed researchers to formalize biological assumptions and explore how changes in one physiological component could influence downstream pregnancy outcomes (14).

For example, models of uteroplacental blood flow can help explain how impaired spiral artery remodeling may reduce placental perfusion and contribute to placental hypoxia. Models of fetal growth can be used to describe expected growth trajectories and identify deviations that may indicate fetal growth restriction. Similarly, placental transport models can provide insight into nutrient exchange and oxygen delivery. Such models have strong mechanistic value because they are grounded in biological principles rather than purely statistical associations.

Nevertheless, virtual pregnancy models have several limitations. Many rely on generalized parameters derived from population averages rather than individual patient data. They may simulate physiological processes accurately at a conceptual level but lack sufficient personalization for clinical decision-making. In addition, traditional virtual models are often static or semi-static, meaning that they do not continuously incorporate new patient-specific information as pregnancy progresses. This limits their ability to reflect real-time changes in maternal or fetal condition (14, 15).

Digital twin technology addresses some of these limitations by introducing a dynamic relationship between the physical patient and the computational model. A digital twin is not merely a simulation; it is a continuously updated virtual representation of a real-world entity. In obstetrics, this means constructing a patient-specific model of pregnancy that evolves as new data become available. The digital twin may incorporate baseline characteristics, longitudinal clinical measurements, imaging data, laboratory biomarkers, molecular profiles, and wearable-derived physiological signals.

The conceptual distinction between virtual pregnancy and digital twin obstetrics is therefore important. Virtual pregnancy emphasizes simulation of pregnancy biology, whereas digital twin obstetrics emphasizes individualized, data-updated prediction and decision support. A virtual pregnancy model may describe how placental insufficiency affects fetal growth in general. A digital twin model, by contrast, would attempt to estimate how placental function, maternal blood pressure, angiogenic markers, and fetal growth trajectory are changing in a specific pregnant individual (16–19).

This dynamic structure creates the possibility of predictive and prescriptive obstetrics. Prediction refers to estimating future risk, such as the probability of developing preeclampsia before 34 weeks or requiring insulin therapy for gestational diabetes. Prescription refers to simulating the likely effect of interventions, such as aspirin prophylaxis, dietary modification, glucose monitoring frequency, corticosteroid timing, or planned delivery. At present, fully operational obstetric digital twins are best described as an aspirational goal rather than a routine clinical tool. Partial or enabling systems exist, including risk-prediction models, longitudinal monitoring platforms, and decision-support algorithms, but these systems should not automatically be labeled as digital twins unless they maintain a patient-specific model that is recalibrated as new data arrive (17–19).

For this review, a pregnancy model is considered to approach a digital twin only when it is individualized, longitudinally updated, able to integrate the modalities needed for the clinical question, linked to the physical patient through repeated data assimilation, clinically interpretable, and designed to support a specific decision or simulate plausible management scenarios. This threshold helps distinguish a static risk calculator from a true patient-linked digital representation (17–19).

## 3 Multimodal data integration

### 3.1 Clinical data as the baseline layer of prediction

Multimodal data integration constitutes the central methodological foundation of digital twin obstetrics. Pregnancy cannot be adequately represented by a single data type because its physiology spans multiple levels of organization, from molecular signaling to organ function and clinical phenotype. A clinically useful digital twin must therefore integrate information across biological scales, including demographic variables, obstetric history, laboratory markers, imaging features, molecular profiles, and continuous physiological signals (20, 21).

Clinical data remain the most accessible and immediately interpretable source of information. Maternal age, body mass index, parity, ethnicity, family history, prior preeclampsia, chronic hypertension, diabetes, renal disease, autoimmune disorders, blood pressure, and routine laboratory findings are widely used in obstetric risk assessment. These variables are valuable because they are inexpensive, routinely collected, and familiar to clinicians. They also provide essential contextual information that helps anchor more complex data types in real-world practice.

However, clinical variables alone are insufficient for high-resolution prediction. Their main limitation is that they often represent downstream manifestations rather than early biological drivers. For example, hypertension in preeclampsia may appear only after placental dysfunction, angiogenic imbalance, and endothelial injury have already developed. Similarly, abnormal glucose tolerance in gestational diabetes may be detected after metabolic dysregulation has been present for weeks. Therefore, clinical data are necessary but not sufficient. They provide the clinical surface of disease, while other data modalities are needed to capture deeper biological processes (22–24).

### 3.2 Imaging and radiomics for structural assessment

Imaging data provide structural and functional information that cannot be obtained from routine clinical variables. Obstetric ultrasound is central to prenatal care because it allows assessment of fetal biometry, amniotic fluid, placental location, fetal anatomy, and Doppler flow patterns. Uterine artery Doppler measurements, for example, may reflect uteroplacental vascular resistance and have been incorporated into early screening strategies for preeclampsia. Fetal growth patterns can also provide important clues about placental function and fetal well-being (20, 25).

Radiomics further expands the value of imaging by converting medical images into quantitative features. Instead of relying only on visual interpretation, radiomics extracts measures of texture, intensity, shape, heterogeneity, and spatial organization. In placental assessment, these features may reflect subtle changes in tissue architecture, vascularization, infarction, calcification, or inflammatory remodeling. Such features may not be obvious to the human observer but can provide predictive information when combined with clinical and biochemical data. In this sense, radiomics may serve as a bridge between macroscopic imaging and microscopic pathology (25).

The incorporation of imaging into digital twin obstetrics is particularly important because pregnancy complications are often mediated through placental and fetal structural changes. Serial ultrasound examinations can provide longitudinal information on fetal growth velocity, placental appearance, amniotic fluid volume, and Doppler resistance. These repeated measurements allow models to identify not only abnormal values but also abnormal trajectories. A fetus whose estimated weight remains within a normal percentile but shows progressive slowing in growth velocity may still be at risk of placental insufficiency. Thus, imaging contributes not only static measurements but also dynamic patterns (26–28).

### 3.3 Multi-omics data for mechanistic characterization

Multi-omics data add a molecular layer to pregnancy prediction. Genomics can identify inherited susceptibility, transcriptomics can reveal gene expression changes, proteomics can capture circulating protein signals, metabolomics can reflect metabolic pathway activity, and epigenomics can provide information about regulatory changes. These data types are particularly valuable because they may identify disease processes before clinical symptoms emerge. For instance, angiogenic imbalance, inflammatory activation, oxidative stress, endothelial dysfunction, altered lipid metabolism, and placental stress pathways may be detectable at the molecular level before overt disease develops (29, 30).

Despite their promise, multi-omics data pose major analytical challenges. They are high-dimensional, expensive, and often generated from relatively small cohorts. Batch effects, platform differences, sample handling, and population heterogeneity can reduce reproducibility. If not carefully handled, machine learning models trained on multi-omics data may overfit and perform poorly in external populations. Therefore, multi-omics integration requires rigorous preprocessing, feature selection, dimensionality reduction, validation, and biological interpretation. In digital twin obstetrics, omics data should not be viewed as standalone predictors but as mechanistic layers that complement clinical, imaging, and physiological data.

The major value of multi-omics lies in its ability to connect predictive modeling with biological explanation. A model that predicts preeclampsia from clinical variables may be useful, but a model that also identifies placental stress, immune activation, or angiogenic dysregulation is more informative. Such mechanistic insight may support earlier intervention and more targeted risk stratification. In the long term, omics-informed digital twins may help distinguish different subtypes of clinically similar disorders, such as early-onset versus late-onset preeclampsia or metabolically distinct forms of gestational diabetes.

### 3.4 Wearable devices and continuous physiological monitoring

Wearable devices and continuous monitoring technologies introduce another crucial dimension: time. Traditional clinical measurements provide isolated snapshots, whereas wearable devices generate high-frequency longitudinal data. Continuous glucose monitoring can characterize glycemic variability in gestational diabetes. Heart rate variability may reflect autonomic function and stress physiology. Sleep, physical activity, and resting heart rate patterns may provide additional information about maternal metabolic and cardiovascular adaptation. These data are particularly relevant to digital twin systems because they allow risk estimates to change as the patient’s physiological state changes (31–33).

The integration of continuous data also changes the nature of prediction. Instead of producing a fixed risk score at one gestational age, a digital twin can generate a dynamic risk trajectory. For example, a patient’s risk of hypertensive disease may be recalculated as blood pressure trends, biomarker levels, and fetal growth measurements evolve. Similarly, a gestational diabetes model may adjust risk classification based on daily glucose variability and response to dietary intervention. This dynamic approach is closer to real clinical reasoning, where decisions are repeatedly updated as new information becomes available (34, 35).

Wearable-derived data also provide an opportunity to move obstetric care beyond clinic-based assessment. Many physiological changes occur between scheduled visits and may be missed by conventional monitoring. Continuous or near-continuous monitoring could help detect gradual deterioration, poor glycemic control, reduced activity, abnormal sleep patterns, or cardiovascular stress before formal clinical diagnosis. However, wearable data must be interpreted cautiously because signal quality, device accuracy, patient adherence, and data overload remain important concerns.

Recent pregnancy-specific wearable studies illustrate this transition from episodic measurement to longitudinal physiology. In a large cohort using commercial wearable devices, Bruce et al. characterized maternal heart rate, resting heart rate, activity, and sleep trajectories across pregnancy and showed that wearables can capture individualized deviations from expected physiologic adaptation. Ravindra et al. used deep representation learning on actigraphy-derived physical activity and sleep data to identify patterns associated with prematurity, while wearable heart-rate-variability studies have explored autonomic markers for preterm birth risk. These studies remain prediction or monitoring models rather than full digital twins, but they provide important longitudinal inputs for future twin systems (36–38).

### 3.5 From data accumulation to integrated pregnancy representation

Multimodal integration is technically demanding because different data types vary in scale, frequency, completeness, noise structure, and clinical meaning. Imaging data are spatial, omics data are high-dimensional, wearable data are temporal, and clinical data are often structured but incomplete. Successful integration requires careful data harmonization, missing-data handling, temporal alignment, normalization, and validation. Without these steps, multimodal models may appear powerful in development datasets but fail in real-world clinical settings.

Overall, multimodal data integration is not simply the accumulation of more variables. Its purpose is to construct a biologically meaningful and clinically interpretable representation of pregnancy. The value of multimodal integration lies in combining complementary information: clinical variables provide context, imaging provides structure, omics provide mechanism, and continuous monitoring provides temporal dynamics. Together, these data streams form the foundation for digital twin obstetrics (Table 1). This rationale is consistent with network physiology, which views health and disease as emergent properties of interacting organ systems rather than isolated measurements, and with multimodal machine-learning reviews showing that clinical, imaging, physiologic, and molecular streams can contribute complementary representations when their timing, scale, and missingness are handled explicitly (39–41).

**Table 1:** Major data modalities for digital twin obstetrics.

Data modality	Representative variables	Main contribution	Key limitation
Clinical data (12, 13, 20–24)	Age, BMI, parity, blood pressure, obstetric history	Direct clinical relevance and easy availability	Limited biological depth
Imaging data (25–28)	Ultrasound, Doppler, MRI, placental morphology	Structural and functional assessment	Operator and protocol variability
Radiomics (25–28)	Texture, intensity, shape, spatial heterogeneity	Quantitative imaging biomarkers	Need for standardization
Multi-omics (29, 30)	Genomics, transcriptomics, proteomics, metabolomics	Mechanistic insight and early molecular signals	High dimensionality and reproducibility issues
Wearable data (31–33, 36–38)	Glucose, heart rate, activity, sleep, HRV	Dynamic longitudinal monitoring	Noise, missingness, and adherence issues

## 4 Modeling strategies

Methodological choices determine whether multimodal pregnancy data can be translated into a clinically useful digital-twin framework. Transparent statistical models remain important benchmarks, but individualized obstetric modeling also requires fusion strategies that preserve modality-specific structure, algorithms that can capture nonlinear and longitudinal patterns, temporal alignment on a gestational-age axis, interpretable outputs, privacy-preserving multicenter validation, and clear links to clinical decisions (12, 13, 40–42).

### 4.1 Traditional statistical models and their limitations

The development of digital twin obstetrics requires modeling strategies capable of handling heterogeneous, high-dimensional, longitudinal, and partially missing data. Traditional statistical models, such as logistic regression and Cox regression, remain important because they are interpretable, transparent, and familiar to clinicians. They are particularly useful when sample sizes are modest and the number of predictors is limited. However, they may struggle to capture nonlinear interactions, complex temporal patterns, and high-dimensional multimodal relationships.

Traditional models also tend to depend on predefined assumptions about variable relationships. For example, linear models may assume that a predictor has a constant effect across the population, whereas in pregnancy the effect of a risk factor may vary according to gestational age, baseline health status, placental function, and coexisting disease. This limitation does not make conventional models obsolete, but it indicates why they are often insufficient for digital twin applications. They may serve as transparent benchmarks, but more flexible modeling strategies are usually required for multimodal prediction. When such models are used, they should still be developed and reported according to prediction-model standards, with attention to calibration, risk of bias, external validation, and clinical utility (12, 13).

An important conceptual distinction that is not always made explicit in the literature is the difference between aggregated (population-level) and personalized (individual-level) modeling. Aggregated models are trained on cohort data and estimate group-level risk patterns; they are designed to perform well on average across a population but may not reflect individual-specific physiology or disease trajectories. Personalized models, by contrast, are calibrated to an individual’s baseline characteristics and progressively updated as new patient-specific measurements become available. In practice, personalization in obstetric digital twins can be viewed as a spectrum rather than a binary distinction. A realistic pathway begins with model pretraining on large, diverse population datasets to learn generalizable physiological patterns, followed by individual calibration using baseline maternal characteristics, obstetric history, and early-pregnancy biomarkers, and then continuous recalibration as longitudinal clinical, imaging, biochemical, and wearable-derived data accumulate throughout gestation. Methodologically, this can be achieved through Bayesian updating, transfer learning, patient-specific fine-tuning, or mixed-effects frameworks that combine fixed population effects with individual random effects. Recognizing where a given modeling strategy falls on this spectrum is essential for evaluating its suitability for digital twin applications.

### 4.2 Fusion level: early, intermediate, late, and hybrid integration

Fusion level refers to where information from different modalities is combined, not to the specific algorithm used afterward. Early or feature-level fusion first transforms clinical variables, biomarkers, radiomic features, omics signatures, and wearable summaries into a shared feature table before fitting a single model. This strategy is simple and often practical for structured datasets, but it can obscure modality-specific noise, timing, and measurement structure (40, 41).

Intermediate fusion combines learned representations before final prediction. For example, an imaging encoder may generate a placental feature embedding, a sequence encoder may summarize glucose or blood-pressure trajectories, and a tabular encoder may represent baseline clinical risk. These embeddings can then be joined in a shared prediction layer. Intermediate fusion is attractive for digital-twin obstetrics because it allows each modality to be processed in a form appropriate to its structure while still producing an integrated patient representation (40, 41).

Late or model-level fusion trains separate modality-specific models and combines their outputs through stacking, weighted averaging, Bayesian updating, or other ensemble approaches. A clinical model may estimate baseline susceptibility, an imaging model may estimate placental dysfunction, an omics model may estimate molecular pathway activation, and a wearable model may estimate current physiologic instability. Hybrid systems can combine early, intermediate, and late fusion. Thus, the key distinction is the integration point, whereas algorithms such as logistic regression, random forest, or XGBoost may be used within any of these fusion designs (40, 41).

### 4.3 Algorithm families for multimodal prediction

Algorithm choice is separate from fusion level. Fusion level determines when and how information from different modalities is integrated, whereas algorithm choice refers to the specific predictive model used after or during this integration process. Therefore, tree-based methods such as random forests, gradient boosting machines, XGBoost, and LightGBM are not limited to a single fusion strategy. They can be applied to early-fused tabular data that combine clinical characteristics, laboratory indicators, imaging-derived features, and other structured variables. They can also be used as modality-specific models in a late-fusion ensemble, or as downstream classifiers for structured representations generated by upstream encoders (22–24, 34).

These algorithms are widely used in clinical prediction because they can capture nonlinear relationships, model complex interactions among variables, and handle mixed data types effectively. In obstetric prediction, tree-based models have been applied to preeclampsia, gestational diabetes, preterm birth, and other pregnancy complications, reflecting their suitability for multifactorial clinical conditions. However, their performance should not be interpreted only by apparent discrimination results. The clinical usefulness of these models remains dependent on dataset quality, population representativeness, external validation, and calibration of predicted risks (35, 40, 41).

Machine learning also allows risk prediction to move beyond single-threshold thinking. Instead of classifying a biomarker as normal or abnormal based on one cutoff, a model can incorporate the biomarker’s continuous value, its interaction with other variables, and its change over time. This is particularly relevant in pregnancy, where many physiological values shift across gestational age. A risk factor that is concerning at one gestational stage may be less meaningful at another. For this reason, model development should make the clinical prediction time point explicit and should avoid mixing measurements from different gestational windows without appropriate temporal encoding (42).

### 4.4 Deep learning for imaging and longitudinal data

Deep learning approaches offer additional possibilities, especially when imaging and time-series data are involved. Convolutional neural networks can extract spatial patterns from ultrasound or magnetic resonance images. Recurrent neural networks, long short-term memory networks, and transformer architectures can model longitudinal sequences such as blood pressure trends, glucose profiles, or fetal growth trajectories. Hybrid models may combine imaging networks, sequence models, and structured clinical data layers into a unified predictive system. Placental MRI radiomics and wearable actigraphy studies illustrate how modality-specific encoders may capture structural or behavioral patterns that are difficult to summarize with single manually defined variables (25–28, 37).

However, deep learning also introduces important problems. These models usually require large datasets and are vulnerable to overfitting when sample sizes are small. Their internal decision-making processes can be difficult to interpret, creating barriers to clinical adoption. In obstetrics, where decisions may affect both mother and fetus, clinicians need to understand why a model assigns high risk. A prediction that cannot be explained may have limited practical value, even if its statistical performance appears strong (43–45).

### 4.5 Explainable artificial intelligence, causal reasoning, and clinical trust

Explainable artificial intelligence is therefore essential, but post-hoc explanation should not be confused with causal inference. Methods such as SHAP values, feature attribution, permutation importance, partial dependence plots, and attention visualization can help identify which variables contribute most strongly to a prediction. These tools may reveal, for example, that a model’s prediction of preeclampsia risk is driven by uterine artery Doppler indices, PlGF levels, mean arterial pressure, and prior obstetric history. Such interpretability allows clinicians to judge whether model outputs are biologically plausible and clinically actionable, but it does not prove that changing the highlighted variable would change the outcome (46).

In clinical settings, interpretability is not only a technical preference but also a requirement for trust. A model that produces a high-risk prediction without explanation may be difficult to use in counseling or decision-making. Conversely, a model that identifies recognizable risk drivers can support shared decision-making between clinicians and patients. Explainable modeling may also help detect algorithmic errors, data leakage, or spurious correlations before deployment. If digital twins are expected to support intervention choices, causal approaches such as directed acyclic graphs, structural causal models, target-trial emulation, counterfactual prediction, and individualized treatment-effect modeling will be needed to estimate what may happen under alternative management strategies. Obstetric examples of target-trial emulation, including comparisons of gestational diabetes diagnostic criteria, show how causal designs can complement prediction models when randomized trials are unavailable or impractical (46, 47).

### 4.6 Temporal alignment and longitudinal multimodal fusion

Another important modeling issue is temporal alignment. Pregnancy data are collected at different gestational ages and at different frequencies. Blood pressure may be recorded repeatedly, ultrasound may be performed at scheduled visits, biomarkers may be measured only once or twice, and wearable data may be collected continuously. A digital twin model must align these streams on a gestational-age axis so that the timing of each observation is preserved. A biomarker measured at 11 weeks has a different clinical meaning from the same biomarker measured at 28 weeks, and a wearable-derived trend may need to be summarized over days or weeks rather than treated as a single isolated value (42).

Practical approaches include storing all measurements with gestational-age timestamps, normalizing variables against gestational-age-specific reference ranges, summarizing high-frequency streams within clinically meaningful windows, using missingness indicators or masks, and applying models designed for irregularly sampled clinical time series. Multimodal temporal fusion can then combine modality-specific encoders: for example, an imaging trajectory encoder for serial fetal growth, a wearable encoder for daily physiologic signals, and a tabular encoder for clinical context. These steps are required before a model can update an individual risk trajectory reliably (36–38, 42).

### 4.7 Federated learning and privacy-preserving modeling

Federated learning addresses a different challenge from temporal alignment. In federated learning, models are trained across multiple institutions without transferring raw patient data; each site trains locally and model parameters or updates are aggregated centrally. This can support multicenter obstetric model development while reducing privacy and data-sharing barriers. However, federated learning alone does not solve the longitudinal modeling problem. Participating sites would still need harmonized gestational-age definitions, time-stamped data structures, missing-data handling, modality-specific encoders, and explicit multimodal temporal fusion before federated training could support a clinically meaningful obstetric digital twin (19, 42, 48, 49).

### 4.8 Clinical utility beyond algorithmic performance

External validation is equally important. Many published obstetric prediction models show promising performance in development cohorts but have limited generalizability when applied to new populations. Differences in ethnicity, healthcare systems, laboratory assays, imaging protocols, disease definitions, and clinical management can all affect model performance. A model developed in one hospital may not perform equally well in another. Therefore, multicenter validation and calibration are essential before clinical implementation.

Ultimately, modeling strategies in digital twin obstetrics must balance predictive performance, interpretability, scalability, and clinical usefulness. A technically sophisticated model is not automatically clinically valuable. For a model to be useful, it must answer a clinically meaningful question, perform reliably in external populations, provide interpretable outputs, and fit into real obstetric workflows. The goal is not to replace clinical judgment but to provide a dynamic decision-support layer that enhances individualized care (Table 2).

**Table 2:** Modeling strategies in digital twin obstetrics.

Strategy	Application	Advantage	Limitation	Modeling scope
Logistic regression (12, 13)	Baseline clinical risk prediction	Transparent and interpretable	Limited nonlinear modeling	Aggregated
Random forest (22–24, 34, 35)	Structured clinical and biomarker data	Handles nonlinear interactions	Moderate interpretability	Aggregated
Gradient boosting/XGBoost (22–24, 34, 35)	Multimodal tabular data	Strong predictive performance	Risk of overfitting	Aggregated
CNN (25–28)	Imaging and radiomics	Captures spatial features	Requires large labeled datasets	Aggregated → Personalizable
RNN/Transformer (37, 42)	Longitudinal physiological data	Models temporal trajectories	Complex and less interpretable	Personalizable
Federated learning (19, 48, 49)	Multicenter model training	Protects privacy and improves scale	Technical and governance complexity	Enabling infrastructure
Explainable AI and causal modeling (46, 47)	Clinical decision support; causal evaluation of intervention scenarios	Improves trust and transparency	May not fully explain complex models	Modeling scope

## 5 Clinical applications

### 5.1 Preeclampsia: dynamic prediction of placental and vascular dysfunction

The clinical value of digital twin obstetrics lies in its potential to support earlier detection, dynamic risk stratification, and individualized intervention for pregnancy complications. Among current applications, preeclampsia has received substantial attention because it is common, clinically serious, and potentially preventable when high-risk individuals are identified early. Preeclampsia is a heterogeneous syndrome involving abnormal placentation, angiogenic imbalance, endothelial dysfunction, immune activation, and maternal cardiovascular maladaptation. This complexity makes it particularly suitable for multimodal prediction (20, 21).

Traditional preeclampsia prediction based only on maternal history and clinical risk factors has limited sensitivity. More advanced screening approaches incorporate mean arterial pressure, uterine artery Doppler, pregnancy-associated plasma protein A, placental growth factor, and other biochemical markers. These approaches reflect the principle of multimodal integration: risk prediction improves when maternal characteristics are combined with markers of placental function and vascular adaptation. A digital twin model could extend this approach by updating risk throughout pregnancy as blood pressure, biomarkers, fetal growth, and symptoms evolve (4, 20, 22).

In clinical practice, such a model could support more individualized monitoring. A patient with rising blood pressure, declining angiogenic markers, abnormal uterine artery Doppler, and slowed fetal growth could be flagged as having a worsening trajectory, even before severe clinical criteria are met. Conversely, a patient with stable physiology might avoid unnecessary surveillance or intervention. This dynamic risk assessment could help guide aspirin prophylaxis, visit frequency, laboratory testing, fetal monitoring, and delivery planning.

### 5.2 Gestational diabetes mellitus: from early risk stratification to real-time management

Gestational diabetes mellitus is another important application. Current diagnosis often depends on oral glucose tolerance testing at 24–28 weeks, but metabolic risk may be present much earlier. Machine learning models using age, body mass index, family history, previous GDM, fasting glucose, lipid profiles, and other metabolic variables can support earlier risk stratification. The addition of continuous glucose monitoring provides a more detailed picture of glycemic variability than isolated glucose measurements. This is especially useful because two individuals with similar diagnostic test results may have very different daily glucose patterns (31–35).

A digital twin model for gestational diabetes could integrate baseline metabolic risk with real-time glucose trends, diet, activity, weight gain, and treatment response. Rather than classifying patients only as having or not having GDM, the model could estimate how glycemic control is changing and whether dietary therapy is sufficient. It could also help identify individuals likely to require pharmacological therapy. Such individualized monitoring may improve maternal glycemic control and reduce risks such as fetal overgrowth, neonatal hypoglycemia, cesarean delivery, and later maternal metabolic disease.

### 5.3 Preterm birth: modeling a heterogeneous syndrome

Preterm birth remains one of the most difficult outcomes to predict because it is not a single disease pathway. Spontaneous preterm birth may involve infection, inflammation, cervical remodeling, decidual hemorrhage, uterine overdistension, stress biology, or premature activation of parturition pathways. Medically indicated preterm birth may result from preeclampsia, fetal growth restriction, placental insufficiency, or maternal disease. Because of this heterogeneity, prediction based on a single marker is unlikely to be sufficient (2, 50, 51).

Multimodal approaches may improve preterm birth prediction by combining obstetric history, cervical length, inflammatory markers, microbiome profiles, transcriptomic signals, uterine activity, and maternal symptoms. A digital twin framework could represent preterm birth risk as a changing trajectory rather than a fixed probability. For example, a patient with a prior spontaneous preterm birth, progressive cervical shortening, inflammatory biomarker changes, and uterine activity may require closer monitoring or intervention. In contrast, stable longitudinal patterns may suggest lower immediate risk (52–55).

### 5.4 Fetal growth restriction and placental insufficiency

Fetal growth restriction and placental insufficiency are also well suited to digital twin modeling. These conditions often evolve gradually and may be reflected in fetal growth velocity, Doppler changes, placental morphology, maternal vascular markers, and angiogenic profiles. Traditional assessment frequently relies on estimated fetal weight percentiles, but growth trajectory may be more informative than a single measurement. A digital twin model could integrate serial fetal biometry, Doppler patterns, placental imaging, maternal blood pressure, and biomarkers to estimate placental reserve and risk of deterioration (25–28, 50, 51).

This approach could be especially useful in distinguishing constitutionally small fetuses from fetuses with true placental insufficiency. Current clinical assessment often depends on percentile cutoffs, but fetal size alone may not adequately represent fetal condition. A multimodal digital twin could incorporate growth velocity, Doppler resistance, maternal vascular status, and placental biomarkers to generate a more individualized risk estimate (26–28).

### 5.5 Decision support for timing of delivery

Digital twin obstetrics may also contribute to labor and delivery decision-making. For high-risk pregnancies, one of the most difficult clinical questions is when to deliver. Early delivery may reduce maternal risk but increase neonatal complications associated with prematurity. Delayed delivery may benefit fetal maturity but increase the risk of stillbirth, severe maternal disease, or emergency delivery. A dynamic model that integrates maternal condition, fetal status, gestational age, and predicted disease progression could support more individualized timing decisions.

Such decision support would be particularly relevant in preeclampsia, fetal growth restriction, and placental insufficiency, where management often involves balancing maternal and fetal risks. A digital twin would not replace clinical judgment, but it could provide structured estimates of likely disease progression under different management scenarios. This could help clinicians communicate risk more clearly and make more transparent decisions with patients.

### 5.6 Current gap between prediction models and true digital twins

Despite these promising applications, clinical implementation remains limited. Most available models are not yet true digital twins in the strict sense. Many are risk prediction models that use machine learning but do not incorporate continuous updating, intervention simulation, or bidirectional feedback. Therefore, it is important to distinguish between current machine learning models and future digital twin systems. The field is moving toward digital twins, but fully validated clinical digital twins in obstetrics remain an emerging goal rather than routine practice (8–10, 56, 57).

This distinction is important because overstating current capabilities may lead to unrealistic expectations. A true obstetric digital twin should be individualized, longitudinal, multimodal, continuously updated, clinically interpretable, and capable of supporting specific decisions. At present, most studies address only one or two of these requirements. Future research should therefore focus not only on improving predictive accuracy but also on building integrated systems that can operate safely and meaningfully within clinical workflows (Table 3). The gap persists because many pregnancy datasets are fragmented across electronic records, imaging systems, laboratories, biobanks, and consumer devices; sample sizes are often too small for high-dimensional longitudinal modeling; and prospective studies rarely collect all modalities at harmonized gestational windows (19). Device and assay heterogeneity, limited external validation, uncertain clinical workflows, regulatory expectations, privacy governance, and the lack of implementation trials further limit progress from prediction models to operational digital twins (36–42, 58).

**Table 3:** Clinical application scenarios of digital twin obstetrics.

Pregnancy complication	Key data inputs	Potential digital twin function	Clinical value
Preeclampsia (4, 20–30)	Blood pressure, uterine artery Doppler, PlGF, sFlt-1, fetal growth	Dynamic risk prediction and disease trajectory monitoring	Early prevention, surveillance planning, delivery timing
Gestational diabetes mellitus (31–35, 47)	BMI, fasting glucose, OGTT, CGM, diet, activity	Real-time glycemic risk modeling	Personalized diet, glucose monitoring, therapy adjustment
Preterm birth (37, 38, 52–55)	Obstetric history, cervical length, inflammation markers, microbiome	Prediction of changing preterm birth risk	Early intervention and targeted monitoring
Fetal growth restriction (25–28, 50, 51)	Serial fetal biometry, Doppler, placental imaging, angiogenic markers	Placental reserve and fetal deterioration assessment	Distinguishing small but healthy fetuses from true FGR
Placental insufficiency (25–28, 50, 51)	Doppler indices, placental morphology, biomarkers, maternal vascular status	Simulation of deterioration risk	Optimized fetal surveillance and delivery planning

## 6 Discussion

The development of digital twin obstetrics represents a major conceptual shift in maternal–fetal medicine. It moves the field away from static, population-level risk prediction and toward individualized, dynamic, and biologically informed modeling. This shift is necessary because pregnancy complications are not uniform events. They arise from complex interactions among maternal physiology, placental function, fetal development, genetics, environment, and healthcare context.

The most important strength of digital twin obstetrics is its ability to integrate information across time and biological scale. In routine practice, clinicians already think dynamically: they interpret blood pressure trends, fetal growth patterns, laboratory changes, symptoms, and gestational age together. A digital twin system formalizes this process computationally, potentially allowing more precise and consistent risk assessment. It may also detect subtle patterns that are difficult for clinicians to recognize manually.

However, several barriers must be addressed before digital twin obstetrics can become clinically reliable. First, data heterogeneity remains a major problem. Obstetric data are collected across different hospitals, devices, populations, and clinical protocols. Ultrasound measurements depend on equipment and operator expertise. Laboratory biomarkers may vary across assays. Electronic health records may contain missing or inconsistent information. Wearable data may be noisy or incomplete. Without careful standardization and harmonization, models may learn site-specific patterns rather than generalizable biological signals (11–13). These concerns are amplified in multimodal systems because each modality has its own sampling frequency, preprocessing pipeline, uncertainty structure, and potential source of bias (36, 40–42). Microbiome-focused causal work, including studies of human skin microbiota and vaginitis, further shows why reproductive-health models may need to account for biologically distinct and site-specific data layers rather than relying only on routine clinical variables (59).

Second, model validation is often inadequate. Internal validation is not enough. A model that performs well in the dataset where it was developed may fail in a different population. Obstetric models should undergo external validation across multiple institutions and should be evaluated not only by discrimination metrics such as AUC, but also by calibration, clinical utility, decision-curve analysis, and impact on outcomes. A model with high AUC but poor calibration may mislead clinicians by overestimating or underestimating risk (12, 13, 43–45).

Third, interpretability is essential. In obstetrics, model outputs can influence decisions about surveillance, medication, hospitalization, and delivery timing. Clinicians need to understand why a model produces a certain prediction. Patients also need explanations that are understandable and ethically acceptable. Explainable artificial intelligence should therefore be integrated from the beginning rather than added as an afterthought.

Fourth, digital twin systems must be clinically actionable. A model that predicts high risk without suggesting what to do next may create anxiety without improving care. For implementation, models should be linked to management pathways, such as aspirin prophylaxis, glucose monitoring, dietary counseling, fetal surveillance, specialist referral, or delivery planning. The clinical question should guide the model design. Prediction alone is insufficient; the goal is better decision-making.

Fifth, ethical and privacy concerns are substantial. Pregnancy data involve both maternal and fetal information, and digital twin systems may combine sensitive clinical, genetic, behavioral, and continuous monitoring data. Clear consent processes, secure data storage, bias assessment, and equitable access are necessary. Algorithmic bias is especially important because obstetric outcomes differ across racial, socioeconomic, and geographic groups. A model that performs poorly in underserved populations could worsen existing disparities (56, 57, 60). Privacy-preserving learning can reduce the need to pool raw data, but it does not remove the need for governance agreements, transparent model evaluation, subgroup performance reporting, patient-facing communication, and safeguards against inappropriate surveillance or overmedicalization (19, 48, 49).

Finally, the success of digital twin obstetrics will depend on interdisciplinary collaboration. Obstetricians, maternal–fetal medicine specialists, data scientists, engineers, bioinformaticians, ethicists, and patients must contribute to model development and evaluation. Digital twins should not be designed as purely technical products (Table 4). They must be clinically meaningful, biologically plausible, ethically acceptable, and usable in real-world care.

**Table 4:** Challenges and future directions for clinical translation.

Challenge	Current problem	Potential solution	Relevance to clinical translation
Data heterogeneity (11–13, 36, 40–42)	Different hospitals use different protocols and devices	Standardized data collection and harmonization	Improves model generalizability
Limited external validation (12, 13, 43–45)	Many models are tested only internally	Multicenter prospective validation	Confirms real-world reliability
Model interpretability and causality (46, 47)	Deep models may behave as black boxes	Explainable AI and feature attribution	Increases clinician trust
Privacy concerns (19, 48, 49)	Pregnancy data include sensitive maternal and fetal information	Federated learning and secure governance	Enables multicenter collaboration
Workflow integration (19, 43–45, 58)	Prediction outputs may not guide action	Link models to clinical pathways	Converts prediction into decision support
Algorithmic bias (19, 56, 57, 60)	Models may perform unevenly across populations	Bias auditing and subgroup validation	Supports equitable care

## 7 Future directions

Future research should prioritize several directions. First, large prospective longitudinal pregnancy cohorts are needed, with standardized clinical data, imaging, biospecimens, and continuous monitoring data collected across gestation. Pregnancy-specific transcriptomic and wearable studies show the value of longitudinal data, but future cohorts must link these modalities within the same individuals and clinical decision windows (29, 30, 36). Second, multimodal data standards and interoperable pipelines should be developed so that clinical, imaging, omics, and wearable features can be harmonized across sites rather than assembled as *ad hoc* datasets (37, 40, 41).

Third, models should incorporate biological knowledge rather than relying only on data-driven pattern recognition. Mechanistic constraints, gestational-age-specific reference ranges, and network-physiology concepts may improve plausibility and reduce overfitting. Fourth, explainable and causal modeling should be emphasized. SHAP and related approaches can support clinical interpretation, whereas causal diagrams, structural causal models, target-trial emulation, and counterfactual prediction are needed to evaluate potential interventions. Fifth, federated learning and privacy-preserving analytics should be explored for multicenter collaboration, but only alongside explicit temporal alignment and harmonization procedures (39, 42, 46–49).

Finally, clinical trials and implementation studies should evaluate whether digital-twin-supported care improves outcomes, reduces unnecessary interventions, improves patient experience, or supports shared decision-making. The field should also avoid overclaiming (8–13). Digital twin obstetrics is promising, but most current studies remain early-stage and many are better described as machine-learning prediction models, monitoring platforms, or digital-twin-enabling components rather than complete twins (17–19). A mature obstetric digital twin should be individualized, longitudinal, multimodal, continuously updated, clinically interpretable, and capable of supporting decisions. This remains an ambitious but achievable goal (43–45, 58).

## 8 Conclusion

From virtual pregnancy to digital twin obstetrics, maternal–fetal medicine is entering a new stage of data-driven precision care. Virtual pregnancy models provide mechanistic insight into maternal–placental–fetal physiology, while digital twins offer the possibility of individualized, dynamic, and continuously updated prediction. By integrating clinical variables, imaging, radiomics, multi-omics, and wearable-derived physiological data, digital twin systems may improve early detection and personalized management of pregnancy complications (8–10, 56, 57).

Nevertheless, digital twin obstetrics remains an emerging field. Its clinical translation will require standardized data infrastructures, robust external validation, explainable modeling, ethical governance, and integration into real clinical workflows. If these challenges can be addressed, digital twin obstetrics may shift pregnancy care from reactive management toward proactive, individualized, and mechanism-informed intervention (11–13, 58, 60).

## Glossary

AI: artificial intelligence
AUC: area under the receiver operating characteristic curve
BMI: body mass index
CGM: continuous glucose monitoring
CNN: convolutional neural network
EHR: electronic health record
EOPE: early-onset preeclampsia
FGR: fetal growth restriction
GDM: gestational diabetes mellitus
HRV: heart rate variability
LightGBM: light gradient boosting machine
LSTM: long short-term memory
MAP: mean arterial pressure
MFM: maternal–fetal medicine
ML: machine learning
MRI: magnetic resonance imaging
PAPP-A: pregnancy-associated plasma protein A
PE: preeclampsia
PlGF: placental growth factor
SASP: senescence-associated secretory phenotype
SHAP: Shapley additive explanations
XGBoost: extreme gradient boosting
